# Can Exclusion of Feral Ecosystem Engineers Improve Coastal Floodplain Resilience to Climate Change? Insight from a Case Study in North East Arnhem Land, Australia

**DOI:** 10.1007/s00267-024-01940-2

**Published:** 2024-02-15

**Authors:** Daniel R. Sloane, Emilie Ens, Yumutjin Wunungmurra, Lanydjana Mununggurr, Andrew Falk, Richard Wunungmurra, Goninyal Gumana, Gillian Towler, Dave Preece, The Yirralka Rangers

**Affiliations:** 1 School of Natural Sciences, Macquarie University, North Ryde, Sydney, NSW 2109 Australia; 2 The Yirralka Rangers, Laynhapuy Homelands Aboriginal Corporation, Yirrkala, NT 0880 Australia

**Keywords:** Cultural ecosystem, Cross-cultural ecology, Indigenous engagement, Buffalo, Pig, Protected areas

## Abstract

Global climate change can interact with local drivers, such as ecosystem engineers, to exacerbate changes in ecosystem structure and function, with socio-ecological consequences. For regions of Indigenous interest, there may also be cultural consequences if species and areas affected are culturally significant. Here we describe a participatory approach between the Indigenous (Yolngu) Yirralka Rangers and non-Indigenous researchers that explored the interaction between sea level rise and feral ungulate ecosystem engineers on culturally significant floodplains in the Laynhapuy Indigenous Protected Area (IPA), northern Australia. A feral ungulate exclusion fence array (12 fenced and 12 unfenced plots) was stratified by elevation/salinity to disentangle the effects of salinity and ungulates on floodplain soil and vegetation. We found that exclusion of feral ungulates improved ground cover vegetation, which, according to our literature-derived ecosystem process model, may enhance soil trapping and reduce evapotranspiration to provide the antecedent conditions needed to improve floodplain resilience to sea level rise. The mid-zone of the supratidal floodplain study site was suggested as the region where the benefits of fencing were most pronounced after two years and ground cover species diversity was highest. Ongoing monitoring is required to investigate whether removal of feral ungulates can increase resilience against sea level rise and recruitment of eco-culturally significant *Melaleuca* species. An interview with a key Yolngu Traditional Owner of the study site demonstrated the importance and effectiveness of the partnership. Yolngu land owners and rangers were active co-researchers and will decide if, when and how to integrate results into feral ungulate management and climate adaptation responses, highlighting the importance of industry-university partnerships in maximising biocultural conservation outcomes.

## Introduction

Global factors, such as sea level rise, can interact with coastal local drivers to cause structural and functional ecosystem changes (Saintilan and Rogers 2015). One local driver that has been observed to interact with sea level rise and exacerbate the rate of landward saltwater intrusion is the presence of animal ecosystem engineers. For example, in coastal areas of the United States of America, such as Louisiana and Maryland, nutria (*Mycastor coypus*) root herbivory has resulted in soil erosion and conversion of tidal freshwater marshes into open water; whilst removal of the animals lead to successful restoration of marsh ecosystems (Kendrot 2011).

In the Northern Territory of northern Australia, introduced Asian Water Buffalo (*Bubalus bubalis*) and wild pigs (*Sus scrofa*) act as animal ecosystem engineers of coastal ecosystems. Buffalo are suspected to intensify saltwater intrusion into coastal floodplains, floodplain fringe backwaters and adjacent freshwater billabongs due to the creation of swim channels and creek extension (Mulrennan and Woodroffe 1998; Bayliss and Ligtermoet 2017). Due to the shallow waters in northern Australia, sea level rise is occurring at three times the global average rate (White et al. 2014) and may be interacting with feral ungulate degradation to intensify saltwater intrusion (Mulrennan and Woodroffe, 1998). This has resulted in vegetation change from freshwater to more salt tolerant species and is therefore threatening local Indigenous peoples’ culturally significant freshwater hunting grounds (Sloane et al. 2018, 2021). Feral buffalo are influencing culturally significant ecosystems such as *Melaleuca* forest (Bowman et al. 2011, Ens et al. 2017, Sloane et al. 2018), aquatic water lilies (primarily *Nymphaea* spp.) (Ens et al. 2012) and *Eucalyptus* savannahs (Werner and Murphy 1987). So well observed are the bio-hydrogeomorphic effects of buffalo that the eastern Kunwinjku people of West Arnhem Land have incorporated buffalo into their dreaming stories of river formation (Altman, 1982).

Simultaneously, coastal floodplains in the Northern Territory are also under increasing stress from wild pigs (*Sus scrofa*), which are responsible for an average upheaval of 10.9 tonnes of soil per hectare per year in monsoonal zones (Hancock et al. 2017). Pig-induced median global soil carbon emissions, in the non-native range of pigs, has been estimated at 4.9 million metric tonnes annually; the equivalent of 1.1 million average passenger vehicles’ annual emissions (O’bryan et al., 2021). The influence of these feral ungulates has been linked to increased water acidity in lagoon environments (Doupé et al. 2010) and production of acid sulfate soils (Sloane et al. 2018) often with a pH below four (Inraratna et al., 1995), presenting another risk factor for coastal floodplain ecosystems such as *Melaleuca* forests (Sloane et al. 2018, Webb et al. 2016). Pig exclosures have been demonstrated as an effective strategy to supress the influence of these ecosystem engineers, and in Malaysia were observed to increase tree recruitment by 233% compared to pig affected sites (Ickes et al. 2001). Similarly, pig exclosures adjacent to seasonally inundated swamps in North Queensland displayed significantly lower seedling mortality rate within fences (19.2%) than corresponding unfenced plots (27.8%) (Mitchell et al. 2007).

In the Laynhapuy Indigenous Protected Area of north east Arnhem Land, large scale (yet patchy) dieback of culturally significant *Melaleuca* forest was noticed by local Indigenous Yolngu people and attributed to the interaction between sea level rise and ecosystem degradation by feral buffalo and pigs (Sloane et al. 2018). Oxidised soil pH measurements and field observations of jarosite within pig and buffalo wallows within the study region also suggest that feral ungulates are disturbing the buried sulfidic sediments characteristic of Australia’s coastal floodplains, and creating acid sulphate soils (Sloane et al. 2018). This further demonstrates that feral ungulates are likely interacting with sea level rise in complex ways. A recent retrospective study of decade-old, isolated exclusion plots suggested that exclusion of feral ungulates from floodplain fringe environments (where *Melaleuca* spp. often reside) can maintain surface elevation by up to 0.96 cm per year, almost matching the region’s rate of sea level rise (Sloane et al. 2021), and therefore may constitute a viable climate change management approach.

Management of feral ungulates on Indigenous owned-land is further complicated by the often conflicting values of ungulates being food, pets and/or threats (Robinson et al. 2005, Albrecht et al. 2009, Ens et al. 2016). Nevertheless, whilst some studies have suggested impacts on *Melaleuca* forest are being intensified by feral buffalo and sea level rise interactions (Bowman et al. 2011, Sloane et al. 2018, Sloane et al. 2021, Stocker 1970), there is no study we are aware of that was specifically designed to disentangle the effects of feral ungulates and sea level rise on *Melaleuca* forest and coastal floodplain health and recovery in Australia. This is imperative so that land managers can make informed management decisions that maximise conservation outcomes for bioculturally important coastal ecosystems.

**Fig. 1:**
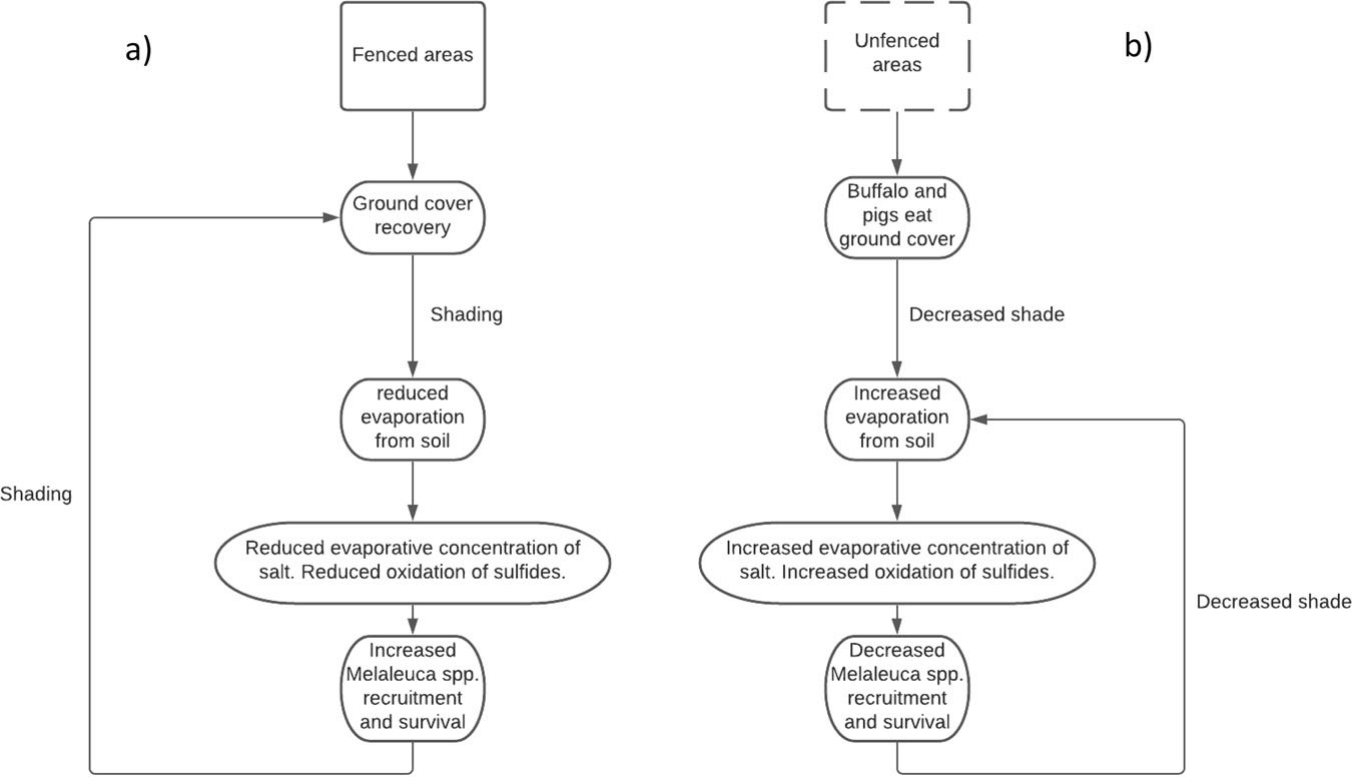
Ecosystem process model for the Melaleuca floodplain/fringe forest: **a** indicates the expected effect of fencing/feral ungulate exclusion from the ecosystem based on existing data and observations; and **b** indicates unfenced or current (buffalo and pig invaded) ecosystem conditions

To inform land manager decision-making for improved ecosystem resilience to climate change, an understanding of the drivers of observed ecosystem decline, such as animal ecosystem engineers and sea level rise, is required. Therefore, a theoretical process model was developed from Traditional Owner observations and Western scientific literature (Fig. 1). This model was derived from the understanding that fenced areas are likely to increase ground cover (Sloane et al. 2021, Ens et al. 2016, Muthoni et al. 2014) which decreases both soil evapotranspiration (Kotanen 1997) and the concentration of surface salts which is likely to facilitate the growth of *Melaleuca* spp. (Vandermoezel et al. 1991), resulting in a feedback shading effect. Here we tested the validity of our proposed ecosystem process model with a participatory action research approach. Yolngu land owners and knowledge holders worked with Macquarie University scientists to establish a feral ungulate exclusion experiment to assess, using robust statistical methods, the interaction between feral ungulates and salinity on floodplain vegetation and soil characteristics. This investigation aimed to answer the question: ‘If feral ungulates (as deleterious ecosystem engineers) can be controlled, would this improve coastal ecosystem resilience to climate change and associated saltwater intrusion into coastal freshwater floodplains?’

## Methods

### Study Site

**Fig. 2:**
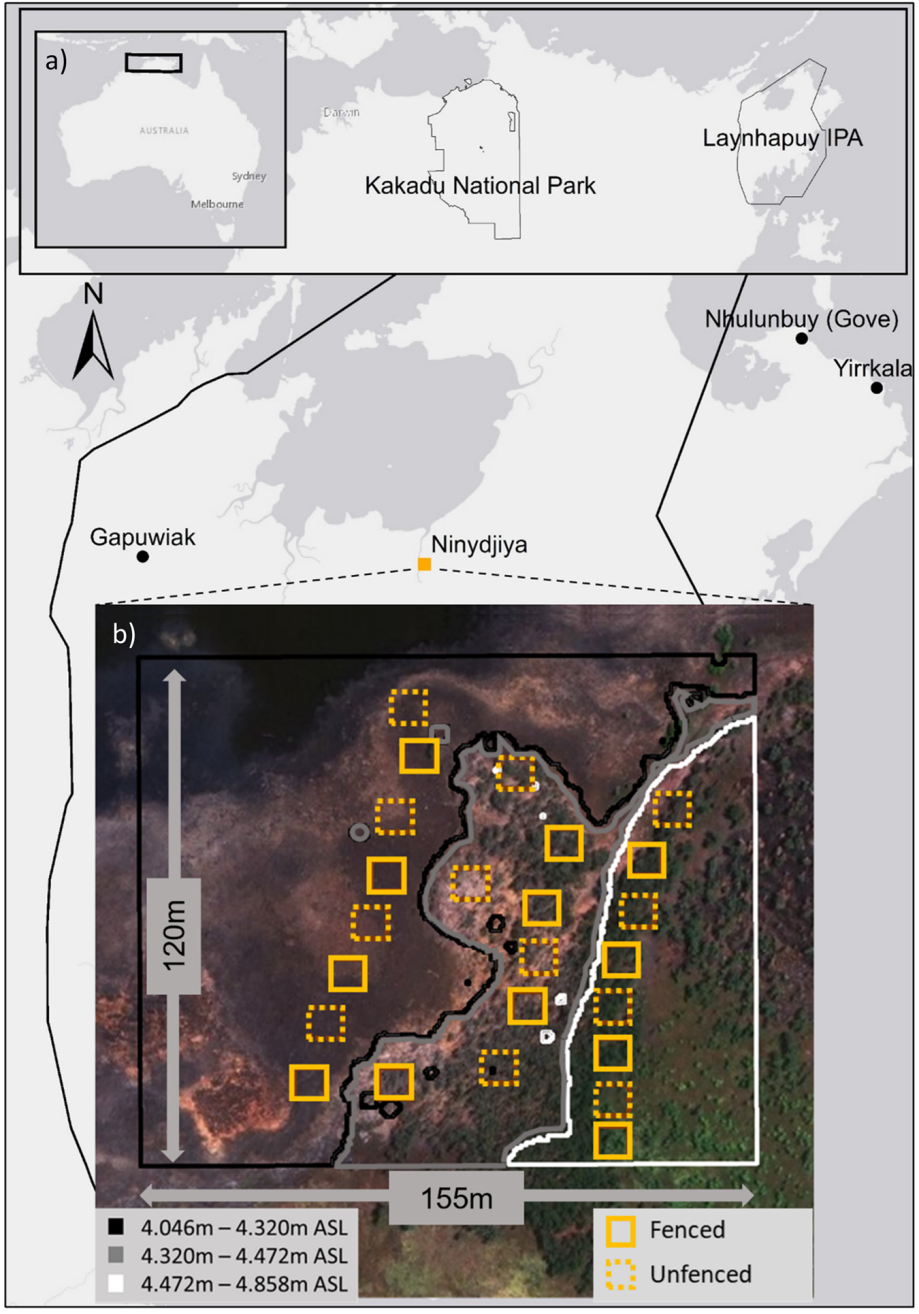
**a** Location of Ninydjiya within the Laynhapuy Indigenous Protected Area in the Northern Territory of Australia, shown with reference to Kakadu National Park; **b** Study site expanded with fenced (solid square) and unfenced (dotted square) plot locations across three elevation levels: black (low), grey (mid) and white (high)

The floodplain study site was located at Ninydjiya (Yolngu word for floodplain) (location formerly called Gurrumuru), a Dhalwangu (a Yolngu clan) homeland of the Laynhapuy Indigenous Protected Area (IPA) of north east Arnhem Land, northern Australia (Fig. 2). University researchers and the Indigenous (Yolngu) Yirralka Rangers worked with the local Yolngu community to carry out the research using a social learning and participatory approach (Keen et al. 2005).

This site was selected through consultation with local Yolngu land owners and previous research that indicated this site was an exemplar of the interaction between soil salinity and feral ungulates potentially impacting on *Melaleuca* species and wetland health (Sloane et al. 2018). Whilst statistical inference regarding other floodplains would have been stronger if multiple floodplains were used, this was not practical given the remoteness of the area, available study sites, the nature of the access points, permissions and community interests. The chosen floodplain hosts sacred sites associated with patches of *Melaleuca* spp. trees and Dhalwangu Traditional Owners requested university scientists to help assess the site partly for this reason. The soil and vegetation characteristics are similar to floodplains across northern Australia (Fitzpatrick et al. 2011, NVIS Technical Working Group 2020), thus results are expected to be generalisable overall despite the practical limitations.

**Fig. 3:**
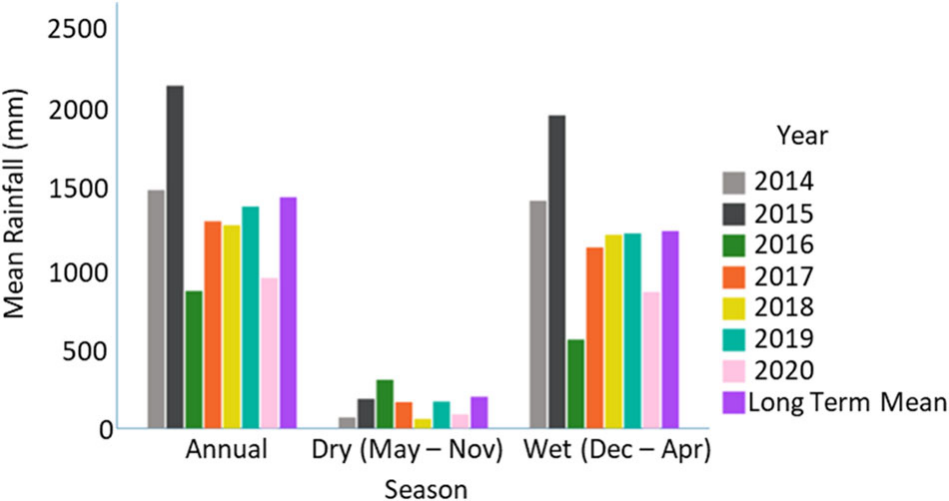
Rainfall at closest weather station (Gove Airport) to the study site in years 2014–2020 (annual, dry season and wet season) compared to the long term mean

Like most of northern Australia, the Laynhapuy IPA experiences a monsoonal climate, with distinct annual wet and dry seasons (Cowie et al. 2000) (Fig. 3), although Yolngu people divide the year into seven seasons (Wettenhall and Preece 2016). Mean monthly rainfall ranges from a minimum of 5.1 mm in August to a maximum of 277.3 mm in January (Fig. 3). Annual mean monthly minimum and maximum temperatures are 22.5 °C and 30.8 °C respectively (The Australian Bureau of Meteorology 2021). In 2016 and 2020, the study site experienced lower than average annual rainfall and wet season (December-April) rainfall (Fig. 3).

### Yolngu Traditional Owner interview

Throughout the experiment, Traditional Owners and university scientists shared knowledge with each other across cultures and strengthened ongoing relationships through participatory co-learning (e.g. Hill et al. 2020). After three years, one of the senior Traditional Owners (and co-author), Yumutjin Wunungmurra, was recorded to tell the story of the work, how the floodplain used to be, and express some of the cultural significance of the area. This was carried out under prior informed consent and Macquarie University Human Ethics approval (reference number 5201500755). The interview was conducted in English (with key words in local Aboriginal language, Yolngu *matha*), ran for one hour and was video recorded. Yumutjin was recompensed through his employment as a Yirralka Rangers cultural advisor.

### Study Site Vegetation and Inundation

The floodplain study site is seasonally inundated, resulting in seasonal variation in the herbaceous ground cover. Ground cover dries off or is visually absent during the dry season and forms near 100% cover during the wet season, largely due to swathes of spike rushes (*Eleocharis* spp.), wild rice (*Oryza sativa*) and other grasses (Cowie et al. 2000). Tall *Eleocharis* species such as *E. dulcis* and *E. sphacealata* are functionally significant species on the floodplain, and provide habitat for magpie geese (*Anseranas semipalmata*), a protected species in all Australian states (Clancy 2020). The corms of *E. dulcis*, locally known as räkay, are an important food source both Aboriginal people and magpie geese. The geese and their eggs are also valuable Aboriginal bush foods (Bayliss and Ligtermoet 2017). The floodplain fringe canopies are dominated by paperbark trees (*Melaleuca* spp.; Cowie et al. 2000) that are inundated by up to 1 metre of water in the wet season, whilst surface water is often absent in the dry season (Finlayson 2005). The biogeography of *Melaleuca* species often reflects patterns of flooding and salinity tolerance (Cowie et al. 2000). Ninydjiya is also a culturally significant site for Yolngu people and hosts sacred sites that are associated with patches of *Melaleuca* trees. Therefore, the investigation of threats to the health of *Melaleuca* forest at this site also had significant cultural importance for local people.

### Experimental Design

In this exclusion experiment two factors were manipulated.

1. Feral ungulate presence (Two levels: fenced and unfenced).
2. Elevation/proxy for salinity since saltwater accesses lower elevations more readily than higher elevations. (Three levels: high, medium, and low).

This yielded six treatment combinations. Each treatment combination was replicated four times, yielding 24 plots of which half were fenced and half were unfenced. Plots were 10 × 10 m squares. To ensure independence, plots were spaced at least 10 m apart.

### Plot Locations

Strategic placement of plots across the low-medium-high elevation points on the floodplains (Fig. 2b) was used to assess the effects of salinity on vegetation and soil. The use of elevation transects followed the US Geological Survey (2012) standard operating procedure, with a high resolution modification to better capture micro-topography (Leong et al. 2018) and coupled relative elevation methods for use under dense canopies. Since there were no continuously operating reference stations (CORS) nearby, we set up a real time kinematic (RTK) base station ca. 150 m away from tree canopies to ensure maximum fidelity in satellite communication. The base was left in position for 24 h to continually correct its own x, y and z values to the point of maximum accuracy thereby producing a temporary elevation benchmark. We then utilised the RTK rover to capture elevation points along the floodplain. Elevation points were captured every metre of visual change in elevation (whichever was the smaller distance) along the 8 transects upon which the fenced and unfenced plots were to be located. These transects were 200 m long with a NW – SE orientation (same orientation as the salinity gradient). The transects were laterally located 50 m apart. Elevation data was converted to metres above sea level according to Australian Height Datum. This was calculated from the geoid height returned by the RTK GPS and computed using the geoscience Australia ausgeoid converter: https://geodesyapps.ga.gov.au/ausgeoid2020.

**Fig. 4:**
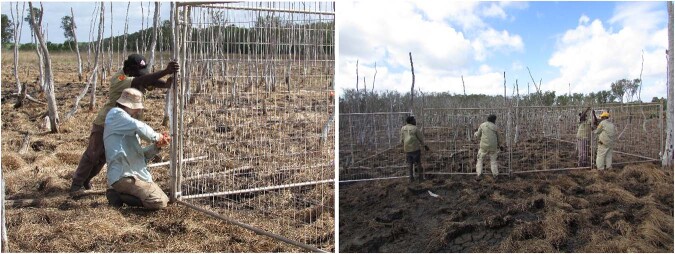
Fence construction during June 2018

Once the elevation map was established, a Jenks classification with three categories was used in Arcmap (v. 10.5) to find the statistical separation between the three elevation levels which were to act as a proxy for salinity. Once the elevation levels were mapped, plot locations were collaboratively chosen with Yolngu land owners as they were able to select locations they wanted to protect and study within the three elevation levels (Fig. 4).

### Fence Construction

Fences were collaboratively constructed with the Yolngu Yirralka Rangers in June 2018 (dry season; Fig. 4). Each fence consisted of 16 panels (each 2.5 m wide; weld mesh purchased from 300Tempfence), with four panels per fence side. The corners and each second join between panels were reinforced with a galvanised star picket, hammered to a depth of one metre, and wired to the panel. Fences were checked regularly and were repaired when necessary.

### Ecological Monitoring

Initial ecological monitoring was performed before fence construction in June 2018 (T0), with subsequent monitoring performed in the early and late dry season thereafter for three years (Table 1). Monitoring was not possible in the early dry season of 2020 due to the COVID-19 pandemic.

**Table 1:** Experiment activities and dates

Month/Year	Activity	Season
June 2018	Elevation survey, fence construction, initial monitoring	Early dry season
November 2018	Second monitoring	Late dry season
June 2019	Third monitoring	Early dry season
October 2019	Fourth monitoring	Late dry season
June 2020	Monitoring not possible (Covid-19)	Early dry season
November 2020	Fifth monitoring	Late dry season

### Soil Chemistry

**Fig. 5:**
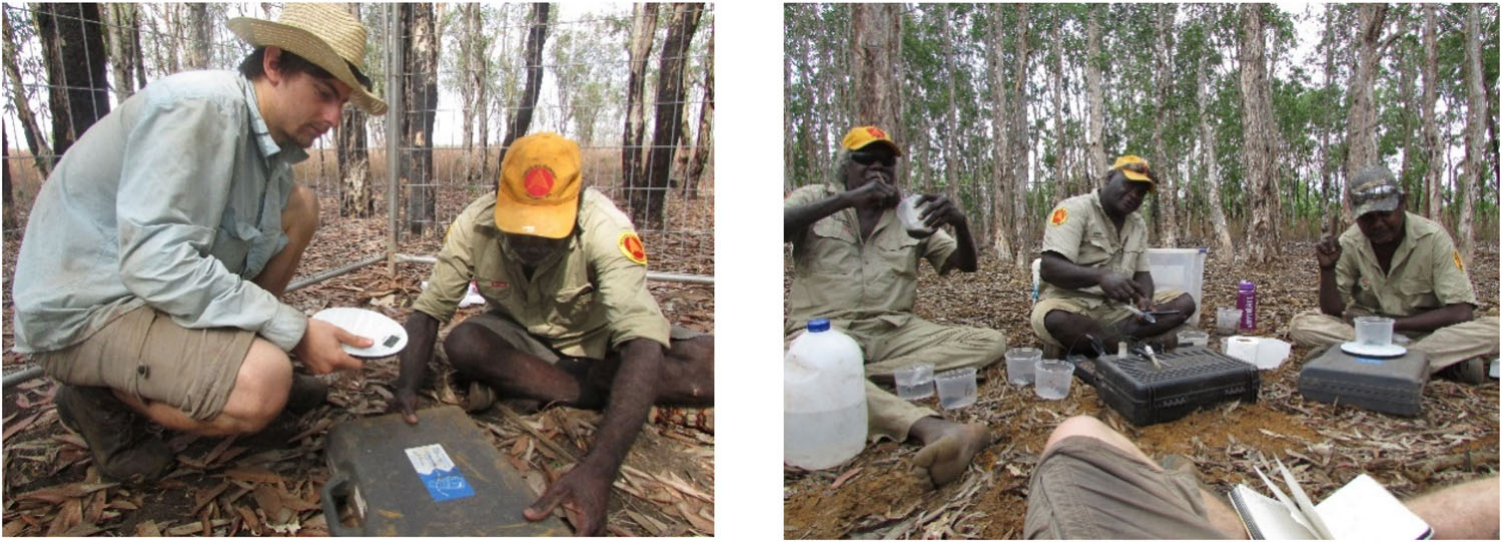
Soil pH monitoring in November 2018

Electrical conductivity (EC) and pH were tested at 10 points along each plot’s corner-corner diagonal, at 10 cm depth. EC was measured using a using a HI993310 Direct Soil Activity and Solution Conductivity metre (Hanna Instruments, Keysborough, VIC, AUS) with a 25 °C automatic temperature compensation, accurate to ±0.4 mS/cm. pH was measured according to Rayment and Lyons (2011; Fig. 5). This required that soil water was squeezed from the soil by hand before 5 g of soil was mixed with 25 mL of demineralised water (soil: water ratio 1: 5). pHw was measured using a PCSTestr35 electronic metre (Thermo Scientific, Waltham, MA, USA), accurate to ±0.01 pH units.

### Ground Cover and Surface Features/Feral Ungulate Damage

Within each of the 24 plots, 1 m point-intercept transects were used to collect ecological data. Ten parallel transects of 10 m each yielded 100 points per plot. Data collection was performed with a purpose-designed CyberTracker® electronic data collection application (Liebenberg et al. 2017). CyberTracker enabled predetermined ‘single choice’ selections similar to Ens et al. (2012 and 2016) for the ground surface feature, ground cover feature and overhead foliage projected cover. Possible choices for ground surface features were: pig damage (tracks or digging), buffalo damage (track or wallow (large circular depression)), flat ground or disturbed ground. Ground cover feature choices were: bare ground, leaf litter, dead wood, water, grass or tree.

### Vegetation

At each point along the point-intercept transects, plant species were recorded (if present), identified and plant height was estimated. At each point the projected foliage cover (presence or absence of overhead leaves) was assessed using a canopy densitometer.

A *Melaleuca* census (combining *M. cajaputi* and *M. viridiflora*) was performed for each plot where individuals were: counted; assigned a health class (healthy, sick or dead) based on level of chlorosis as in Sloane et al. 2018; and height estimated (categories: <10 cm, 10–30 cm, 30–60 cm, 60 cm–1 m, 1–2 m, 2–5 m, 5–10 m or >10 m).

### Statistics

To ensure comparability of fenced and unfenced plots at each elevation, a one way ANOVA with Tukey’s HSD post-hoc test was used (except when homogeneity of variances was violated in which case the Games-Howell statistic was used) to verify that the elevation levels represented unique levels of salinity (as suggested by Leong et al. (2018)) and pH. Assumptions of normality and homogeneity of variance was verified using the Shapiro-Wilk test and Levene’s test, respectively. To minimise the influence of potential pseudo-replication, data was averaged to one value per replicate according to the methods detailed in Lazic et al. (2020). All statistical tests were completed using SPSS (ver. 25.0, IBM,Armonk, NY, USA; see https://www.ibm.com/products/spss-statistics, accessed 12 Jan 2024).

To assess changes in vegetation ground cover and canopy cover, we used a repeated measures ANOVA in which the between subjects factors were elevation (high/mid/low) and fence (presence/absence) whilst the within subjects factor was time. Within subjects effects were used to compare the effect of time and its interaction with elevation and fence on each dependent variable (vegetation ground cover and *Melaleuca* spp. canopy cover), whilst controlling for each plot having a unique quantity of each dependent variable at T0. Mauchly’s test of sphericity verified the assumption of sphericity. If failed, the Greenhouse-Geisser variation was interpreted. For canopy cover, lower plots were excluded due to having no canopy cover for the duration of the experiment.

## Results

### Reflections of a Traditional Owner

**Fig. 6:**
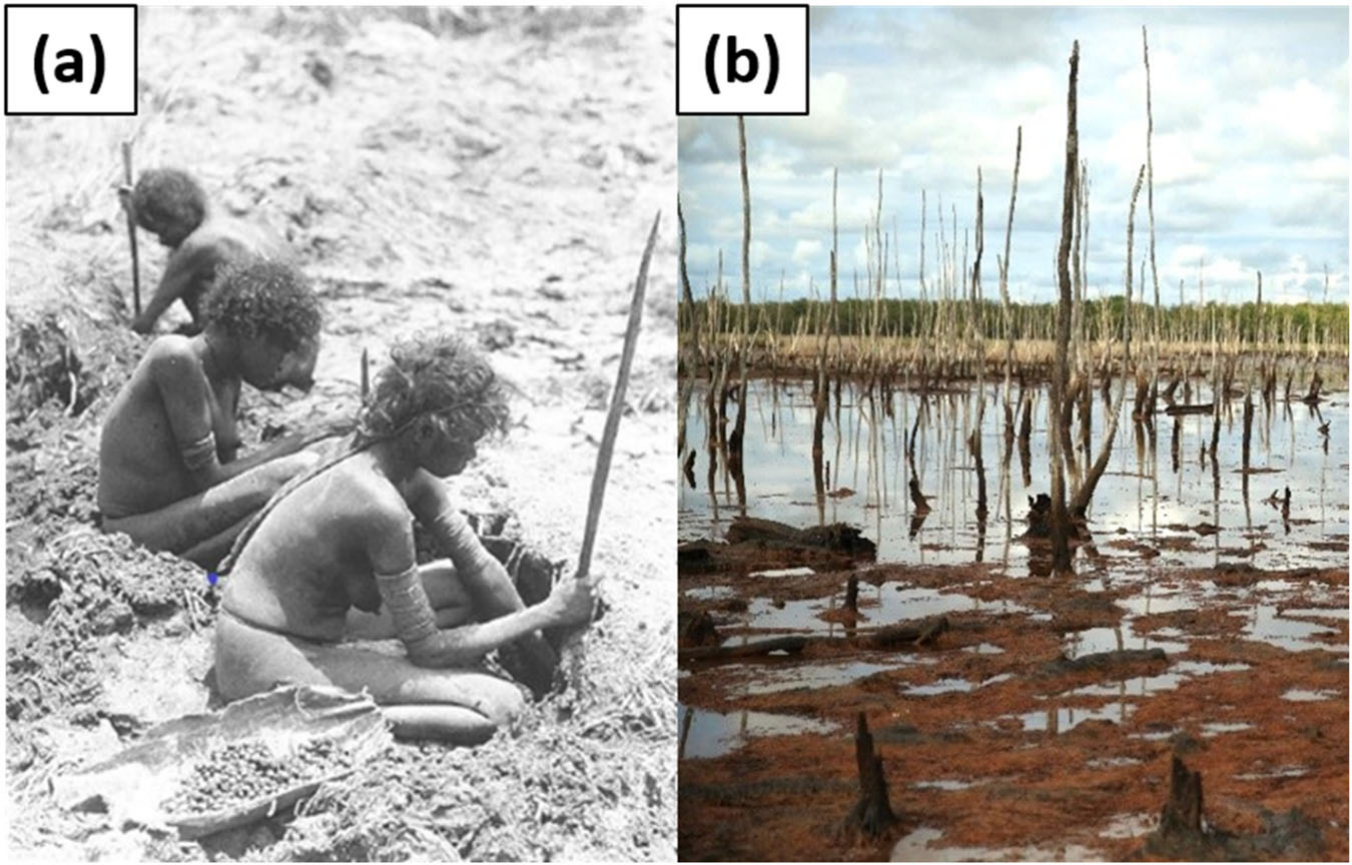
**a** Women using digging sticks to collect räkay (*Eleocharis dulcis*) corms and storing them in *rangan* (paperbark, *Melaleuca* spp.) carry barks. Photo: Donald Thomson 1935 (available in: Thomson and Peterson 2003); **b** Ninydjiya floodplain 2021. Dead standing *Melaleuca* spp., pugged and uprooted *räkay* (*Eleocharis dulcis*) and soil. Photo: Daniel Smuskowitz

Yumutjin Wunungmurra shared his reflections with the team and explained why Ninydjiya and feral ungulate exclusion research was important:

“You can see this place, some of the trees [*Melaleuca* spp.] dying. You can see *monuk gapu* [salt water] coming up. That was a change [compared] to what we’ve seen before. Killing our lives, for Yolngu. Because we’ve got names on this *wanga* (place), songlines and paintings. [It’s] also killing our *ngatha*, bush tucker. We don’t see [any] long-necked turtles: bakarra [*Chelodina* spp.], *nindan*, *dhirrang* [tubers of *Nymphaea* spp.]. That’s our *ngatha* [food] names. More important this place is killing lives for Yolngu, because I can see trees dying. I don’t know. We are doing *djama* [work] here. Using the little machines (soil probes) and checking up and find out what’s [it] all about, *dhawu* [story], from this place. I think you’ve been at another place [other Yolngu homelands], maybe same story or different from this. Our mother’s mob and grandmother’s mob used to get *räkay* [*Eleocharis dulcis* corms] from this Country and *nindan* and *dhirrang*, and *bakarra* [Fig. 6a]. Now, *bayngu* [nothing]. How can we help this place? We’ll try. Try work, really hard work to help this place. Try, we can heal back this *wanga*. Just because our old people [have] been through here. Walking, hunting, get *bakarra*, *räkay*, *nindan*, or any other Yolngu *ngatha*. This time *bayngu*. I’m sitting, I’m watching that country. Lot of digging from *piggy piggy* [pigs], lot of foot track [from] *gadabanga* [buffalo]. Those two animals are killing the Country, and they’re killing animals, *ngatha*. Another one coming up: saltwater. Because those two animals are making a road, when the rain starts the saltwater comes right up to the inland… And people get sick from this… Used to be old ladies sitting on the *dharpa* (trees), but now they are all gone, they die. We’ll try help this Country, with my *gathu* [son]. He’s the one who came from university [first author], he help us doing *djama* here. Really hard work and find out [why the trees are dying] from mud and from salt water. I can see that I feel sick from this Country now.”

### Soil Chemistry

**Fig. 7:**
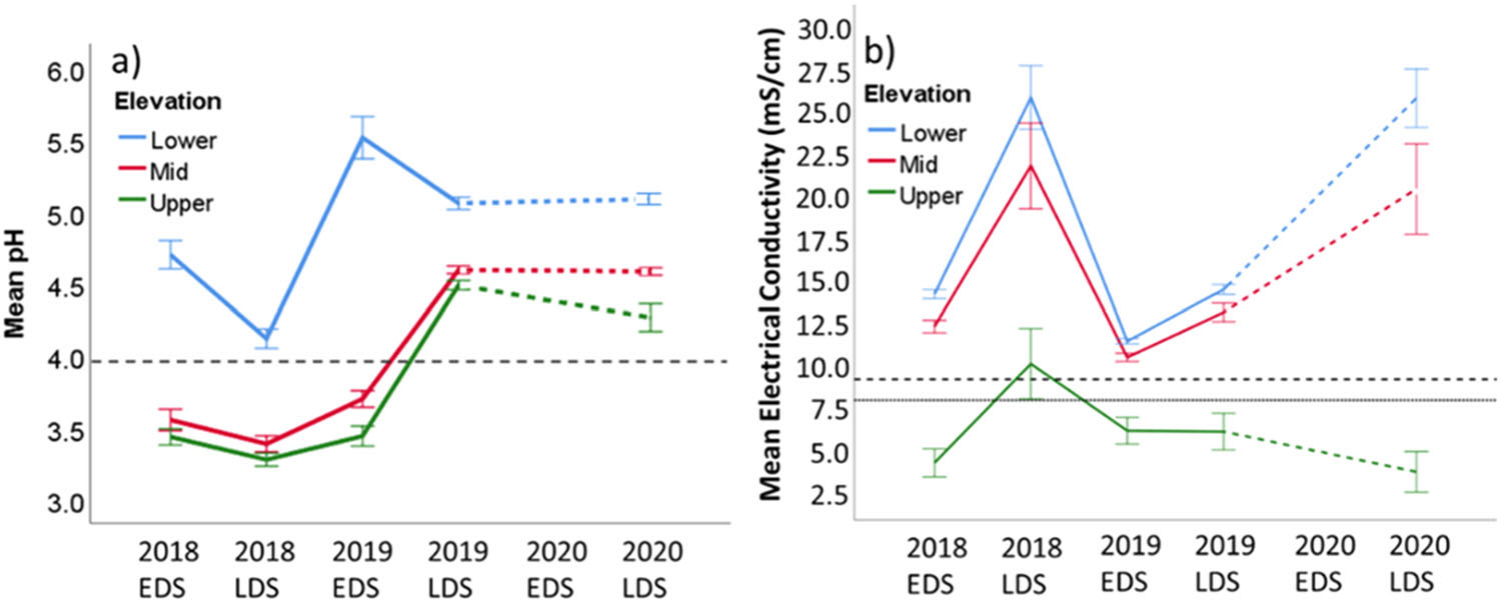
Mean (**a**) pH and (**b**) EC of each elevation level at each time. Error bars represent 1 standard error. The pH reference line (black dash) at pH 4 indicates the presence of acid sulphate soils (Inraratna et al. 1995). The EC dotted and dashed lines indicate the minimum salinity at which dead M. viridiflora (8.08 mS/cm) and M. cajuputi (9.32 mS/cm) respectively, were documented in the Laynhapuy IPA, May 2016 (Yolngu season Dharratharra – Early Dry Season) (Sloane et al. 2018), comparable to times 0, 2 and 4. Fenced and unfenced plots were grouped together as no significant fence effect was found

Upper plots always had a significantly lower salinity (EC) than mid and lower plots, regardless of season or year (Table 2), and largely remained within the salinity tolerance of dominant Melaleuca species (M. cajuputi and M. viridiflora) (Sloane et al. 2018) across the study period, except in the late dry season (LDS) 2018 (Fig. 7b). For the mid and lower plots, soil EC was above the expected salinity tolerance level of these species (Sloane et al. 2018, Moezel et al. 1991). Whilst EC in the mid and lower plots was similar in the LDS, lower plots exhibited significantly higher EC than mid plots in the early dry season (EDS) (Table 2, Fig. 7b).

**Table 2:** One-way ANOVA of pH and electrical conductivity (EC) at each time step. Tukey/Games Howell grouping compares significant mean differences between upper, mid and lower elevation levels within each time step

		pH					Electrical conductivity (EC)				
Time	df	F	*p*	Tukey/Games-Howell			F	*p*	Tukey/Games-Howell		
				Lower	Mid	Upper			Lower	Mid	Upper
EDS 2018	2	79.67	<0.01	A	B	B	92.78	<0.01	A	B	C
LDS 2018	2	64.99	<0.01	A	B	B	13.97	<0.01	A	A	B
EDS 2019	2	128.87	<0.01	A	B	C	33.88	<0.01	A	B	C
LDS 2019	2	76.56	<0.01	A	B	B	39.13	<0.01	A	A	B
LDS 2020	2	45.90	<0.01	A	B	C	34.41	<0.01	A	A	B

Repeated measures ANOVA revealed a significant interaction between time and elevation on EC (F_(4.074,36.664)_ = 8.526, *p* < 0.001). The EC at all three elevation levels initially moved in step for the first three measurement periods, after which the lower and mid elevation EC increased whilst the *upper elevation* EC remained similar or decreased (Fig. 7b). There was no significant interaction between time, elevation and fence (F_(4.074,36.664)_ = 1.678, *p* = 0.175), or time and fence on EC (F_(2.037,36.664)_ = 0.862, *p* = 0.433), indicating no statistically significant fence effect at this stage. The time by elevation by fence cubic contrast was nearly significant (F_(2,18)_ = 3.441, *p* = 0.054), thus the cubic trend lines of EC for this interaction may be starting diverge, indicating that the fences may be starting to have an effect.

During the study period lower plots always had a significantly higher pH than mid or upper plots, regardless of season or year (Table 2) and consistently remained above the acid sulphate soils (ASS) reference line (Inraratna et al. 1995) (Fig. 7a). The soil pH of the mid and upper plots was below the ASS reference line at the start of the study (from the EDS 2018 to the EDS 2019), though pH was greater than this reference line in later measurement periods (Fig. 7a). The upper plots were more acidic than mid plots, and this was significant in the EDS 2019 and LDS 2020 (Table 2). Repeated measures ANOVA revealed a significant effect of time by elevation on pH (F_(4.679,42.114)_ = 27.410, *p* < 0.001). There was no significant interactions for time by elevation by fence (F_(4.679,42.114)_ = 0.407, *p* = 0.830), or time by fence on EC (F_(2.340,36.664)_ = 0.851, *p* = 0.485), indicating that the fences had no statistically significant effect on pH at this stage. Contrast analysis detected no significant trends in pH.

### Feral Ungulate Damage

**Fig. 8:**
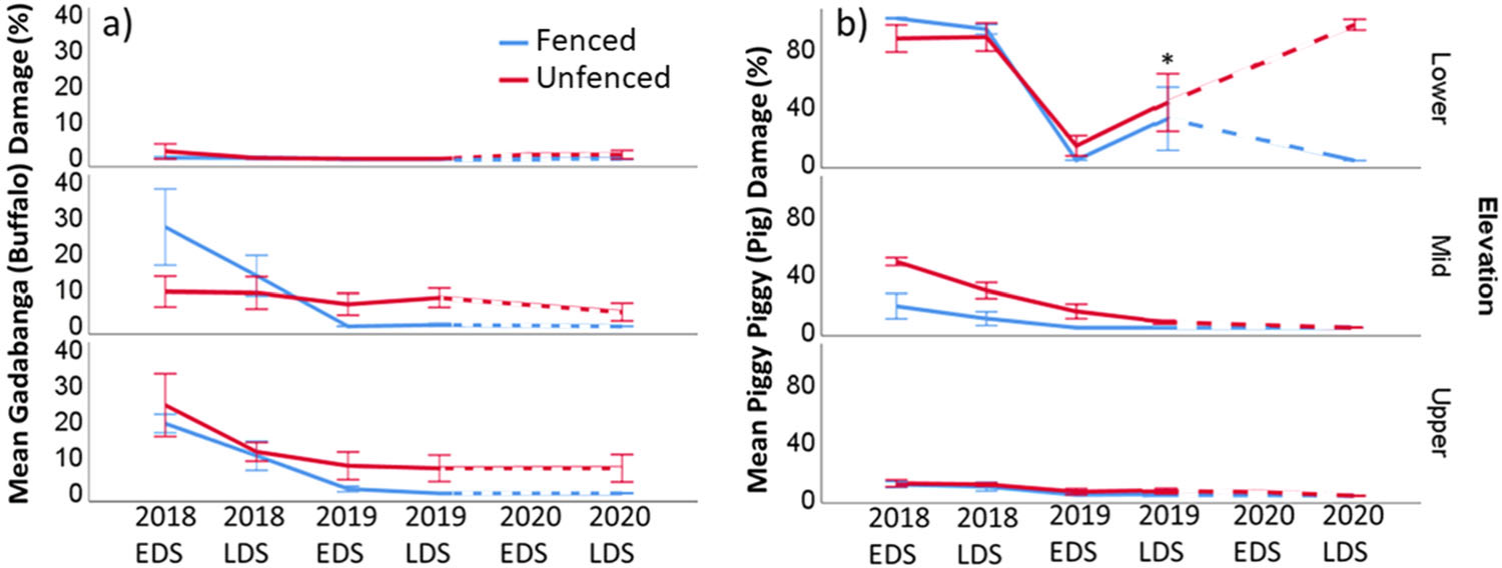
Mean percent (**a**) gadabanga (buffalo) damage and (**b**) pig damage in fenced (blue) and unfenced (red) plots at each elevation over time. EDS Early dry season, LDS Late dry season. Error bars = ±1 SE. *fence breach. Dashed line indicates lack of data from 2020 EDS

*Gadabanga* (buffalo) damage was highest in the upper elevation plots (Fig. 8a). The mid plots had a mix of *gadabanga* and pigs, whilst lower plots were damaged more by pigs (Fig. 8b). Visible *gadabanga* damage decreased after one wet season in the fenced mid and upper elevation plots and low levels of damage were maintained due to *gadabanga* exclusion (Fig. 8a). In the mid and upper unfenced plots, the *gadabanga* damage remained consistent at around 10%, although in the upper plots this was after an initial decrease from 20% at the start of the experiment (Fig. 8a). There was a similar decline in pig damage in the fenced and unfenced mid elevation plots over time, though this was likely due to the loss of räkay (*Eleocharis dulcis*: the preferred food of pigs) at this elevation level (see Fig. 10). Lower elevation fenced plots were breached by pigs in the LDS 2019 (asterisk); however, after successful repair, the pig damage decreased back to zero in one year (Fig. 8b).

### Ground Vegetation Cover

**Fig. 9:**
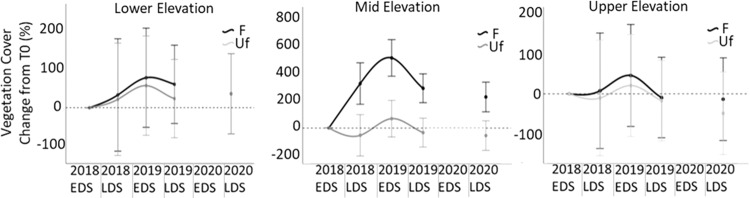
Percentage change in mean vegetation cover from time 0 (T0) in fenced (F) and unfenced (Uf) plots at each elevation level. EDS Early dry season, LDS Late dry season

Ground cover vegetation was significantly different across elevation levels (Between subjects; F_(2,18)_ = 38.777, *p* < 0.001), explaining 81.2% of the variation regardless of the time period (Table 3). Within subjects (plots), time by elevation had a highly significant effect on ground cover (*p* = 0.002; Table 3). Similarly, the contrast detected a significant difference in the slopes of the linear vegetation cover trend lines between elevation levels (F_(2,18)_ = 15.208, *p* < 0.001). Therefore, the vegetation cover of the middle elevation plots showed an uptrend in cover through time and was relatively stable at the upper and lower elevations (Fig. 9). Within subjects, the interaction of time by fence was not significant (*p* = 0.095; Table 3). The time by fence contrast detected a significant difference in the slopes of the linear trend lines between fenced and unfenced plots (F_(1,18)_ = 5.373, *p* = 0.032), suggesting that the fence may be aiding ground vegetation recovery.

**Table 3:** Repeated measures ANOVA for vegetation ground cover

Sources of variation	Type III sum of squares	df	Mean square	F	Sig.	Partial Eta squared
Between subjects						
Intercept	233640.880	1	233640.880	245.916	0.000	0.932
Elevation	73683.050	2	36841.525	38.777	0.000	0.812
Fence	121.866	1	121.866	0.128	0.724	0.007
Elevation * Fence	1126.834	2	563.417	0.593	0.563	0.062
Error	17101.511	18	950.084			
Within subjects						
Time	6530.453	4	1632.613	14.117	0.000	0.440
Time * Elevation	3212.751	8	401.594	3.473	0.002	0.278
Time * Fence	952.869	4	238.217	2.060	0.095	0.103
Time * Elevation * Fence	1240.046	8	155.006	1.340	0.238	0.130
Error(Time)	8326.595	72	115.647			

The assumption of sphericity was met (χ^2^(9) = 5.632, *p* = 0.777)

Fenced plots were associated with a linear uptrend in vegetation cover whilst unfenced plots did not deviate much after T0; however, this result was mostly influenced by increased vegetation cover in the mid elevation (Fig. 9). Time by elevation by fence was not associated with significant change in mean vegetation cover (*p* = 0.238; Table 3), and the contrasts did not detect a significant difference in trend either, with the quadratic contrast being closest to significance (F_(2,18)_ = 2.163, *p* = 0.144). Therefore, the effect of fencing on vegetation cover over time did not significantly differ between elevation levels.

**Fig. 10:**
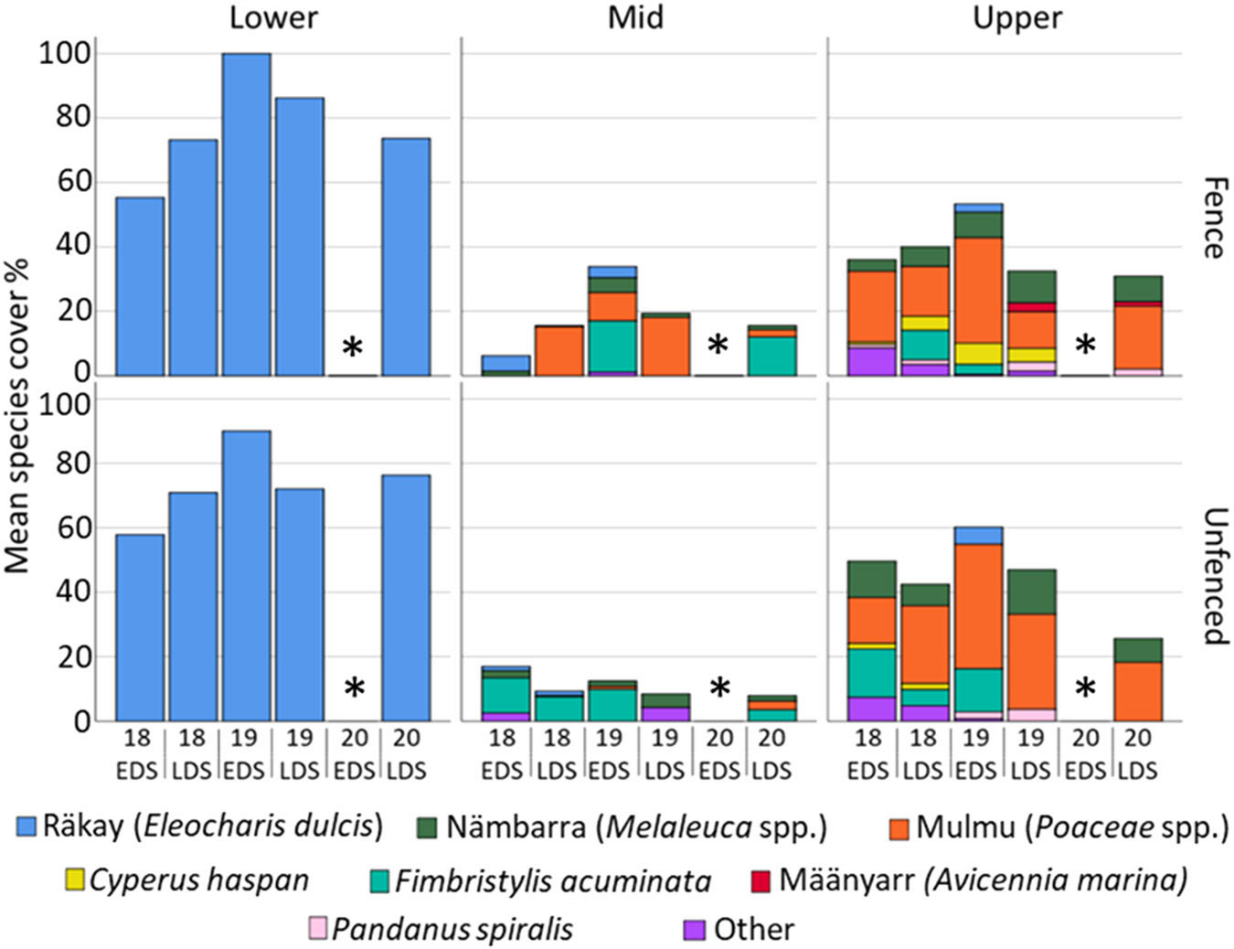
Species cover and diversity in fenced and unfenced plots at lower, mid and upper elevations across 5 time points. * no monitoring due to COVID-19. EDS Early dry season, LDS Late dry season

Lower plots were dominated by a monoculture of *räkay* (*Eloecharis dulcis*), whereas middle and upper plots had a more diverse mix of species (Fig. 10). In the middle elevation, there was a general increase in *mulmu* (*Poaceae* spp.) in fenced plots, whilst unfenced plots remained dominated by *Fimbristylis acuminata*. The upper fenced plots maintained species diversity whilst a decrease in diversity was observed in unfenced plots (Fig. 10).

The between subjects effect of elevation on *Melaleuca* spp. canopy cover was highly significant (*p* < 0.001), explaining 91.6% of the variation regardless of the time period (Table 4). Within subjects, time by elevation was significant, explaining 50.2% of the variance in canopy cover (Table 4). This was due to mid plots declining in canopy cover, canopy cover in upper plots remained stable. There was no time by elevation by fence or time by fence interaction (Table 4), indicating fences had no impact on canopy cover over the study period.

**Table 4:** Repeated measures ANOVA for canopy cover

Sources of variation	Type III sum of squares	df	Mean Square	F	Sig.	Partial Eta squared
Between subjects						
Intercept	135984.518	1	135984.518	176.955	0.000	0.908
Elevation	150614.400	2	75307.200	97.997	0.000	0.916
Fence	834.312	1	834.312	1.086	0.311	0.057
Elevation * Fence	1017.447	2	508.724	0.662	0.528	0.069
Error	13832.412	18	768.467			
Within subjects						
Time	901.168	1.533	587.806	4.403	0.030	0.197
Time * Elevation	3714.582	3.066	1211.458	9.075	0.000	0.502
Time * Fence	8.861	1.533	5.780	0.043	0.922	0.002
Time * Elevation * Fence	69.587	3.066	22.695	0.170	0.919	0.019
Error(Time)	3683.985	27.596	133.498			

Greenhouse–Geisser adjusted statistics reported due to violation of sphericity (χ^2^(5) = 30.878, *p* < 0.001)

### Melaleuca Demography

**Fig. 11:**
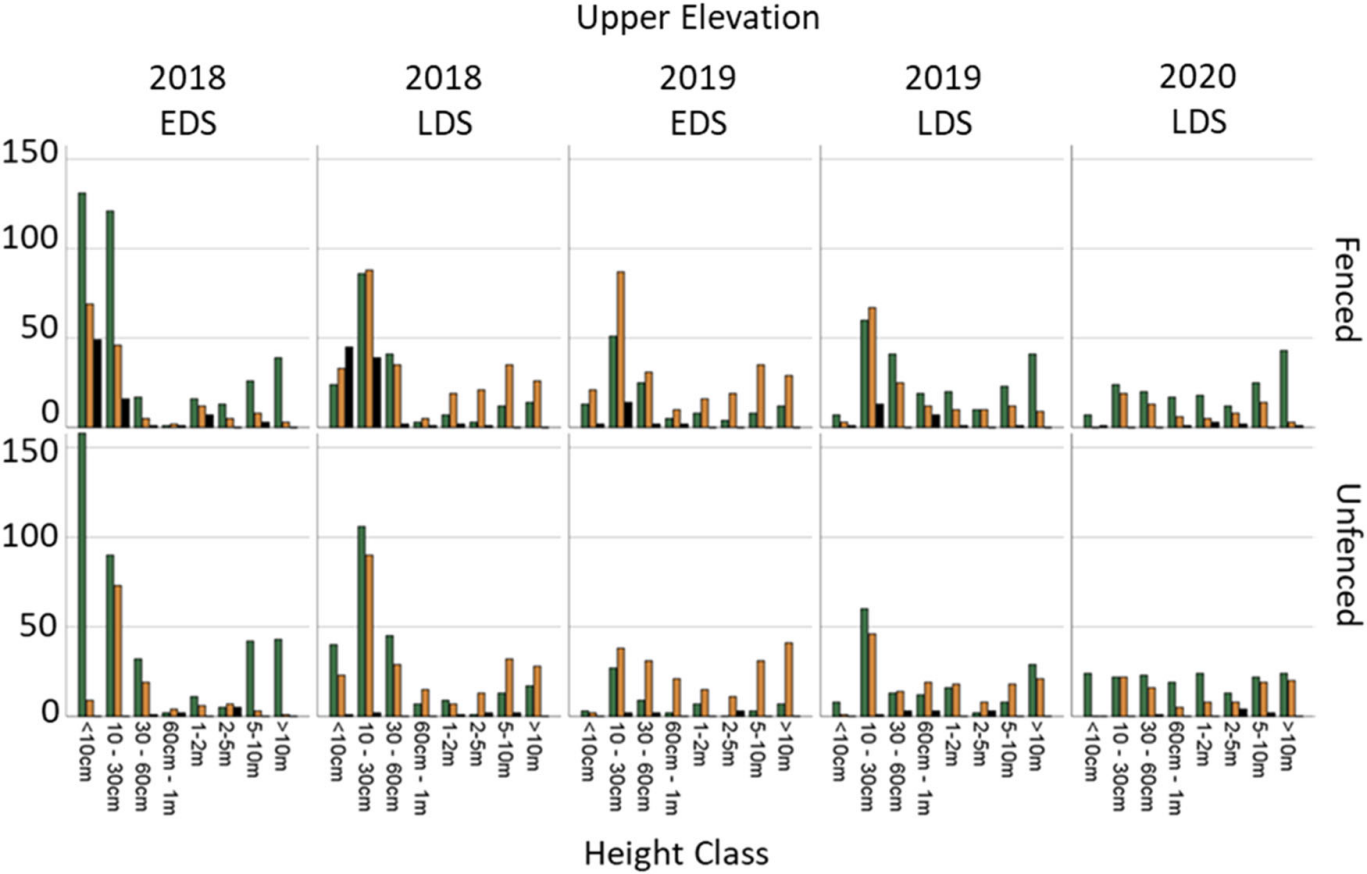
*Melaleuca* species demography in the upper elevation, at fenced and unfenced plots at each time period. EDS Early dry season, LDS Late dry season

*Melaleuca* species only occurred at upper and mid elevations. In the upper elevation there were no major trends in *Melaleuca* species demography between fenced and unfenced plots during the study period. Marginally, there was greater survival of the <10 cm and 10–30 cm classes between the 2018 late dry season (LDS) and the 2019 early dry season (EDS) in fenced compared to unfenced plots. In the 2019 LDS, unfenced plots displayed an increase in the number of 10–30 cm plants, whilst fenced plots exhibited increased progression to the 30–60 cm size class. In both fenced and unfenced plots, throughout the time series, progression to the 2–5 m class was limited. In fenced plots, trees taller than 5 metres appeared to recover, as they moved from the sick to healthy class; especially between the 2019 EDS and 2019 LDS. Concurrently, unfenced plots showed trees in these classes also recovering their health at these times, though they could not match the ratios of healthy to sick trees displayed by the fenced plots (Fig. 11).

**Fig. 12:**
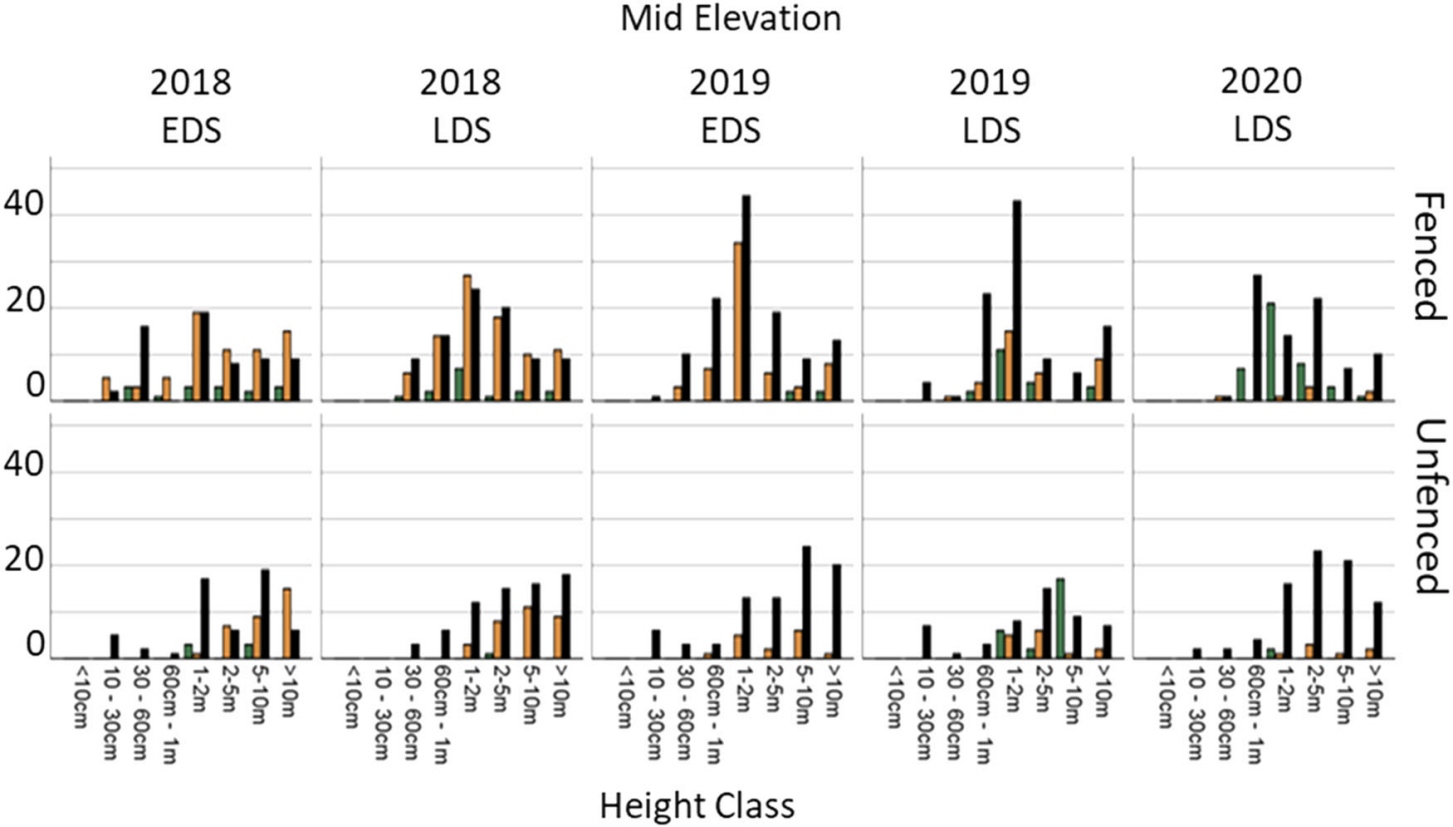
Melaleuca demography in the mid elevation, at fenced and unfenced plots at each time period. EDS Early dry season, LDS Late dry season

In the middle elevation there was little to no recruitment in the small (<1 m) size classes in either the fenced or unfenced plots. 2019 LDS showed an increase in healthy *Melaleuca* species in both fenced and unfenced plots particularly in size classes over one metre. Healthy plants were retained only in the fenced plots by 2020 LDS and were lost in the unfenced plots (Fig. 12).

Most of the ecological change as a result of feral ungulate exclusion fencing occurred in the mid elevation plots, with some effect at the upper elevation level (Table 5). In the lower fenced plots, a reduction in pig damage and a slight increase in *räkay* (*Eleocharis dulcis*) cover occurred (Table 5).

**Table 5:** Summary table of effects of the fence on measured ecological parameters across the three elevations at Ninydjiya floodplain

Green = positive, Orange = no effect, Grey = not applicable

## Discussion

This study utilised a participatory research approach to understand the likely interacting effects of sea level rise and feral ungulate invasion on management-relevant ecosystem processes of a culturally significant coastal floodplain in the Laynhapuy Indigenous Protected Area.

Our results highlighted that the greatest increase in ground cover percentage and species richness from feral ungulate exclusion occurred at the mid-elevation level, suggesting that vegetation here would benefit most from feral ungulate exclusion. This is a significant finding as the mid-elevation level is yet to experience the full impact of sea level rise and associated increases in salinity concentrations which has been fatal for *Melaleuca* spp. at the lower-elevation level. For this reason we suggest that the mid elevation level of the floodplain is most immediately vulnerable to rising sea levels, and without intervention would likely result in the decline in diversity and conversion of remaining *Melaleuca* spp. into a simplified, salinity tolerant *räkay* (*Eleocharis dulcis*) dominated ecosystem. Feral buffalo and pigs also occupied the mid elevation area, while in the low elevation pigs were most common and in the high elevation plots, buffalo were more common. This is in-line with the “multiple stressors” view of forest dieback (Mueller-Dombois 1988), the corollary being that alleviating the stress of contributing factor(s) may promote ecosystem resilience against remaining factors. As such, if feral ungulate exclusion can effectively alleviate excess stress, this offers land managers an opportunity to increase floodplain ecosystem resilience.

Our investigation confirmed the primary hypothesis of our ecosystem process model, where removal of feral ungulates (via exclusion fences) provided the antecedent conditions required to promote ground cover regeneration. Several studies of ungulate exclusion at billabongs (Ens et al. 2016), savannahs (Muthoni et al. 2014), woodlands (Ward‐Jones et al. 2019), monsoon forest (Bowman and McDonough 1991) and floodplains (Sloane et al. 2021) have all documented similar improvements in ground cover vegetation, and therefore, this result was predicted. However, notably, an increase in vegetation as a result of exclusion fences was most prominent at the mid elevation level, suggesting that feral management in this area may promote improved resilience to sea level rise for the most vulnerable region of the floodplain.

The ecosystem process model suggested that an increase in ground cover as a result of ungulate exclusion would provide more shading to the ground, and therefore, reduce evaporative concentration of salts. However, our investigation was not yet able to detect such salinity (EC) improvements in fenced plots over the three-year study period, although there was emerging evidence of a possible late dry season reduction in fenced plot salinity that may become statistically significant with ongoing monitoring. If a reduction in soil salinity and increase in soil moisture were to occur due to shading mechanisms, this may result in a change in species composition away from salt-tolerant species, perhaps to be most pronounced at the mid-elevation level, since soil moisture and salinity are the two most important factors controlling species gradients in coastal wetlands (Moffett et al. 2010, Pettit et al. 2016).

The ecosystem process model also suggested that reduced bioturbation and drying of soils in better vegetated plots would likely result in less oxidation of sulphides and therefore a more neutral pH (Inraratna et al. 1995). However, such changes were not detected over the three years of monitoring. This indicates the need for further long term monitoring such that the increase in ground cover associated with feral exclusion has time to reach a critical threshold. It is also possible that the current method of pH measurement may require refinement as currently it relies on digging up soil which could confound data by oxidising samples prior to measurement. Potential and actual acid sulfate soils have previously been detected at the study site and oxidation of materials is rapid after upheaval (Sloane et al. 2018). The installation of in situ loggers may give a more reliable result.

Whilst *Melaleuca cajuputi* is found in acid sulfate soils within its native range, those soils remain wet the majority of the time (Yamanoshita et al. 2001) and may not exhibit the extremely low pH that can occur in periods of drought in northern Australia (Cowie et al. 2000). Whilst climate projections for northern Australia currently vary on the direction and magnitude of future precipitation trends (Narsey et al. 2020), the 2015/2016 dieback of mangroves (Duke et al. 2017) and ongoing decline of inland forests has been attributed to compounding climate extremes (extremely wet followed by extremely dry periods) and sea level rise in the case of coastal forests, which have no precedent in the last 220 to 250 years (Allen et al. 2021), making vegetation responses uncertain and resilience questionable. Future dry periods would also result in increased oxidation of sulfidic sediments and therefore present a higher acid sulfate risk going forward.

Lastly, the final hypothesis of our ecosystem process model inferred that improved vegetation and soil conditions following feral ungulate exclusion would likely result in increased *Melaleuca* spp. recruitment and survival. Accordingly, we detected that feral ungulate exclusion was improving ground cover vegetation recovery relative to unfenced plots. Again, this result was expected as other studies of ungulate exclusion at billabongs (Ens et al. 2016), savannahs (Muthoni et al. 2014, Werner et al. 2006), woodlands (Ward‐Jones et al. 2019), monsoon forest (Bowman and McDonough, 1991) and floodplains (Sloane et al. 2021) have documented similar improvements. Interestingly, this effect was especially strong in the mid elevation level, suggesting the region of the floodplain ecosystem at the forefront of sea level rise could improve its resilience to sea level rise if feral ungulates are removed. Whilst nascent, it appears that there was improved survival in the ‘less than 30 cm’ size class of *Melaleuca* in fenced plots suggesting that that feral ungulate exclusion may be starting to improve *Melaleuca* spp. survival according to the ecosystem process model.

If feral ungulates are removed from coastal floodplains of the Laynhapuy Indigenous Protected Area (IPA), the changes we detected here on a microscale may extrapolate and confer greater resilience to the IPA as a whole, as hypothesised in our ecosystem process model (Fig. 1). Another two to four years of monitoring the outcomes of this experiment will likely provide robust data to further our understanding of the interacting effects of sea level rise and soil and vegetation change following feral ungulate exclusion. The addition of detailed elevation measurements would also allow further exploration of the hypothesis that feral ungulate exclusion may not only result in a positive feedback loop of soil and vegetation change, but also identify whether erosion and lowering of ground elevation (as suggested by Sloane et al. 2021) may present complementary feral ungulate and sea level rise management opportunities. Mulrennan and Woodroffe (1998) presented buffalo in the list of causes of tidal channel intrusion at Tommycut Creek in Kakadu National Park, and Bowman et al. (2011) undertook a remote sensing analysis indicating an interaction between buffalo swim channels and saltwater intrusion; however, the biogeomorphic impact of these large ungulates has not yet been systematically assessed on-ground to our knowledge.

The present study provided a preliminary exploration into the complex dynamics of in-situ coastal floodplains considering sea level rise and feral ungulates as ecosystem engineers. However, as with all field studies, there are several limitations of the work presented including: the size of the plots, pseudoreplication (we were only able to study one floodplain), limited study period, and grass species identification challenges in the dry season. Nevertheless, early results have already informed local Yolngu management of feral ungulates as they have initiated aerial culls on other floodplains, and helped explain the observed *Melaleuca* spp. dieback, whilst planning and preparing for the possible future of these eco-culturally important species and places.

### Management Implications

This investigation has been underpinned by a collaborative approach between local Traditional Owners and university researchers, in an effort to utilise Western scientific tools to assist Yolngu Traditional Owners in decision-making about management of their ancestral estates. The participatory approach of this project has actively engaged local Traditional Owners in the study conception, design, and data collection process, so they have first-hand knowledge of the results. It is becoming increasingly recognised that models that engage local stakeholders, and focus on place-based values are not only more likely to succeed, but also likely to impact positively on environmental outcomes (Sterling et al. 2017, Deroy and Darimont 2019). This is one key benefit of local participation, rather than scientists simply informing land managers and decision-makers who may be sceptical or not understand scientific results and where they came from (Danielsen et al. 2005). It was clear that the project had cultural importance (see interview with Yumutjin Wunungmurra – results), which was crucial as local “buy-in” from Indigenous groups is key to the success of cross-cultural collaborative conservation projects globally (Schuster et al. 2019). The participatory approach of this investigation provided Traditional Owners with additional tools, experience and knowledge to develop ‘two-way’ understandings that can be further incorporated into local land management decision-making.

Feral animal impact and ecological monitoring research, such as this study, offer management relevant information to assess the effectiveness of feral animal control efforts and identify a range of techniques that could be used to abate impacts (Edwards et al. 2004). The Yirralka Rangers’ knowledge of soil and vegetation change has been heightened and an aerial cull on other floodplains in the Laynhapuy IPA was underway at the time of writing. Since *Melaleuca* spp. are the key affected culturally significant species of the study region (Sloane et al. 2018), and the next stage to be assessed in the ecosystem process model (Fig. 1), we suggest that recruitment and survival of *Melaleuca* spp. be the key indicator of success in the exclusion experiment going forward. Ongoing monitoring of this experiment will inform rangers and Yolngu land owners about whether culling at Ninydjiya floodplain will be enough to protect the culturally significant *Melaleuca* spp. from dying.

If ongoing monitoring suggests that fencing and removal of feral ungulates improve *Melaleuca* spp. outcomes then the Yirralka Rangers may pursue that strategy. Fencing off areas provides immediate benefits visible after just one wet season at billabongs (Ens et al. 2016), and over the longer term on floodplains even when occasional breaches occur (Sloane et al. 2021). Fencing has also been effective on large scales such as Hawaii Volcanoes National Park, where complete eradication of feral pigs was achieved (Katahira et al. 1993) and may prove to be more cost effective than culling without fencing. Fencing also allows for increased success of planting programs as young plants are free of additional pressure on their survival. Fencing also combines well with culling programs as they allow the creation of zones in which effective culling can be achieved without exogenous addition to animal populations (Katahira et al. 1993).

If monitoring suggests that sea level rise is overwhelming and removal of feral ungulates and consequent increased vegetation and trapping of sediment doesn’t facilitate accretion of floodplain elevation enough to prevent significant saltwater intrusion, the Yirralka Rangers and Yolngu Traditional Owners, like Yumutjin Wunungmurra, will need to decide what to do about the *Melaleuca* sacred sites on the floodplain. Will they allow the current course of events to continue and impact on the sites, engage in active protection measures such as culling and replanting, or changing how they use certain sites? Crucially, it will be up to the Yolngu land owners to decide how they wish to integrate science to support their autonomous decision making for themselves and future generations.

## Data Availability

Raw data and associated material are held jointly by Macquarie University and The Yirralka Rangers.
